# Beyond individual integration: Family systems, social support networks and living environment as health determinants among migrants in Germany

**DOI:** 10.1016/j.jmh.2025.100368

**Published:** 2025-10-17

**Authors:** Franziska Reinhardt, Imad Maatouk

**Affiliations:** University Hospital Würzburg, Department of Internal Medicine II, Section of Psychosomatic Medicine, Psychotherapy and Psychooncology, Würzburg, Germany.

**Keywords:** Migration health, Social determinants of health, Family values, Life satisfaction, Health inequalities

## Abstract

While research has established links between migration and health outcomes, the role of cultural and social factors in mediating this relationship remains understudied. This study examines how family relationships, value orientations, and life satisfaction interact with migration status to influence health outcomes in Germany.

Using data from the Family Demographic Panel (FReDA), a representative German study on family relationships, we conducted multiple linear and multivariate regression analyses (*n* = 8939). The study employs a comprehensive set of health indicators, including both objective measures and self-reported health status.

The analyses reveal that migration status alone is not a significant predictor of health outcomes when controlling for social and cultural factors. Instead, life satisfaction and family relationships showed substantial effects on health. Both traditional and modern family values were linked to better health outcomes, showing that stable values, regardless of whether they are traditional or modern, can support health.

These findings challenge simplified assumptions about migration-health relationships and emphasize the importance of recognizing cultural values, social support systems, and psychosocial well-being as relevant factors in addressing health disparities. They also underscore the need for healthcare systems to adopt culturally sensitive approaches that consider the diverse social realities of individuals.

## Highlights

• Family relationships and social support serve as protective health factors, independent of socioeconomic status.
• Both traditional and modern family values contribute positively to health outcomes, suggesting value stability matters more than direction.
• Migration status alone is not a significant predictor of health outcomes when social and cultural factors are considered.
• Social support, particularly within family relationships, serves as a protective health factor.
• Healthcare systems could benefit from family-centered and culturally sensitive approaches.

## 1 Introduction

Migration and related health disparities represent a crucial public health challenge. This is particularly true in countries with significant migrant populations like Germany, where over 26 % of residents have a migration background (Rechel et al., 2013; Destatis, 2023). Although systematic evidence within the German healthcare context is still limited (Böhm et al., 2016; Destatis, 2020). Structural, individual, and policy-related barriers contribute to significant health inequalities for individuals with a migration background even within formally universal healthcare systems (Hacker et al., 2015). Such disparities have been documented across various countries despite differing healthcare structures, highlighting the critical role of national policy environments (Dourgnon et al., 2023).

These disparities are particularly concerning as the health status of migrants is not merely an immigration policy concern, but serves as a critical indicator of health system equity and effectiveness (McDonald and Kennedy, 2004). Germany has a diverse migrant population, which encompasses various cultural, religious, and linguistic backgrounds. However, despite migrants' substantial contributions to Germany's social and economic welfare through tax revenues, social security systems, and cultural diversity (Domnich et al., 2012; Färber and Köppen, 2020), their specific health needs often receive insufficient attention in both research and practice (Rechel et al., 2013).

Current evidence reveals notable health disparities. Migrants in Germany show distinct patterns of healthcare utilization, with lower engagement in preventive care and regular health services, yet higher rates of emergency care usage (McDonald and Kennedy, 2004; Böhm et al., 2016). This pattern, combined with reduced participation in rehabilitation services and elevated rates of early retirement suggests systematic barriers to appropriate healthcare access (Böhm et al., 2016; Van Manh et al., 2020). Moreover, migrants are disproportionately represented among the uninsured population, indicating structural challenges in healthcare accessibility (Böhm et al., 2016).

The relationship between migration and health outcomes is complex and dynamic. While some studies document a "Healthy Migrant Effect" where recent migrants initially show better health outcomes than native populations this advantage typically diminishes over time (Stern, 2020; Coughlin, 2020). Paradoxically, despite generally lower socioeconomic status, some migrant groups exhibit higher life expectancy, suggesting the influence of unmeasured social and cultural factors (Domnich et al., 2012; Böhm et al., 2016). However, this narrative often overlooks the heterogeneity of migrant experiences and the profound health inequalities faced by certain groups. For example, migrants from fragile states such as the Central African Republic (CAR), may carry the long-term health consequences of living in regions with severe public health crises. The CAR is characterized by ongoing instability, an underfunded healthcare infrastructure, and limited access to essential medical services, particularly in rural areas (Green, 2012). These challenges lead to higher rates of untreated infectious diseases, malnutrition, and maternal mortality. Upon migration individuals from such backgrounds often encounter additional structural barriers to access healthcare, exacerbating their pre-existing health conditions and complicating the narrative of the "Healthy Migrant Effect".

### 1.1 Conceptualizing social determinants of health

While these findings underscore the importance of socioeconomic factors in migrant health outcomes, they capture only one dimension of a more complex landscape. To fully understand health inequalities among migrants, it is essential to look beyond economic indicators and patterns of health care use to examine how social relationships, family dynamics and living conditions collectively shape health outcomes. A substantial body of research has repeatedly demonstrated that health is intrinsically linked to our social connections and environment (Holt-Lunstad et al., 2017; Holt-Lunstad, 2021, 2022). Individuals in stable relationships typically show better health outcomes, and strong family ties can serve as crucial resources for maintaining physical and mental well-being (Authors, 2025). Similarly, social support networks and living conditions have been identified as key determinants of health status (Holt-Lunstad, 2024; Kok et al., 2013). However, these social mechanisms have rarely been studied comprehensively in the context of migrant health. This study aims to address this gap by examining how various social determinants influence health outcomes among migrants in Germany. Specifically, we investigate six dimensions of social determinants:

First, we examine basic health disparities between migrants and non-migrants (H1), considering how these differences may vary by socioeconomic status (H1a). Previous research suggests that the reasons for disparities in health outcomes are diverse. A well-researched factor is socioeconomic status. Individuals from lower income brackets often experience poorer health and have less access to healthcare services (Braveman et al., 2011). Moreover, the proportion of people with a migration background is disproportionately high within this group, suggesting that socioeconomic status may act as a moderating factor (Dalsania et al., 2021). Socioeconomic factors can thus be seen as critical determinants for the occurrence of risk factors.

Some studies also show that migration itself impacts health. Pinto de Oliveira et al. (2024) found in their study that health-damaging behaviors increased significantly after arrival in the host country compared to the 12 months prior to departure from their country of origin. However, health disparities are not limited to the time of and around migration period but are observed across the entire lifespan, with disparities becoming more pronounced in old age (Majid et al., 2020). In addition to these individual and migration-specific factors, structural factors such as limited or no access to healthcare services or discrimination-free medical care can negatively affect migrants' health outcomes (Rechel et al., 2013; Meng and Yamauchi, 2017).

Second, we investigate how attitudes toward marriage and family differ between migrants and non-migrants and how this impacts health outcomes (H2). Studies have shown that migration influences attitudes toward family values. Not only does experiencing migration alter value orientations, but individuals who choose to migrate often have different attitudes and values compared to those who remain in their countries of origin (Williams et al., 2014). Their value orientation can be described as transcultural and dynamic, as it is shaped by both the values of their home country and their host country. This dual influence can lead to ambivalence in individual values, balancing between traditional norms and individual freedoms (Williams et al., 2014; Fuchs et al., 2021). Through acculturation processes, value orientations may become more aligned with those of the host society and are often largely adapted in the second generation (Röder and Mühlau, 2014). This complexity makes it challenging to draw clear conclusions about the effects of migration status on family values, as the diversity within migrant communities is significant. It is necessary to consider not only migration status itself but also the cultural background (Andersson, 2021; Fuchs et al., 2021). Studies found that refugees in Germany tendencies towards liberal and democratic values, particularly with respect to gender equality, which were significantly more pronounced compared to the German population (Fuchs et al., 2021). Studies on health and value orientation suggest that traditional value orientations are positively associated with social support, which is considered a crucial factor for mental health (Maercker et al., 2015). At the same time, it becomes evident that the impact of value orientations strongly depends on the social and cultural context. A mismatch between value orientation and context can be regarded as a risk factor for mental health (Maercker, 2016).

Third, we look at the relationship between life satisfaction, happiness, and health outcomes, particularly among migrants (H3). Migrants often evaluate their life satisfaction based on migration-specific factors, which are strongly shaped by their experiences in the host country. Initially, the advantages of migration may appear particularly significant. However, after a while their focus shifts from initial benefits to challenges such as social integration, discrimination, and changing expectations (Hendriks and Bartram, 2019). Studies clearly indicate that legal status, financial security, social networks, and experiences of discrimination are key determinants of migrants' life satisfaction (Paloma et al., 2021). Health plays a central role, as a good state of health is often a prerequisite for experiencing overall well-being. Consequently, social determinants that influence health, such as legal status or financial security, also significantly impact life satisfaction. Migration-related stress and the high demands of adapting to a new environment can lead to tensions, elevated stress levels, and increased vulnerability to mental health issues, which in turn directly affect life satisfaction (Andersson, 2021; Bhugra and Becker, 2005).

Fourth, considering the crucial role of family dynamics in migrant communities, we explore how relationships with parents and intergenerational values affect health outcomes (H4). Evidence suggests that close relationships with parents and the transmission of intergenerational values play a key role in the mental health of migrants, supported by concepts such as cultural identity and social support (Bhugra and Becker, 2005). At the same time, the nature and quality of these relationships are significant. When parents are required to work early or are temporarily absent due to the migration process, this can negatively affect the health of their children. The loss of direct parental care and the high demands placed on families during the acculturation process can have long-term effects on the physical and mental health of the entire family (Meng and Yamauchi, 2017; Haagsman et al., 2015).

Fifth, we examine the role of social support networks in shaping health outcomes (H5). The investigation into the role of social support in health matters clearly shows that social networks act as a critical factor, contributing to better health across all stages of life (Holt-Lunstad, 2022). Migration influences social group affiliations and alters the impact of social support on health. Mao and Zhao (2012) found that the mental health of adolescent migrants is particularly more adversely affected compared to their native peers because they face the developmental task of belonging to peer groups. Social exclusion or the loss of established social networks in the country of origin are further migration-specific challenges. To counter these effects, migrants develop transnational networks that shape their connections to their home countries or turn to new forms of social support, such as religious and cultural communities, which offer a sense of belonging and support (Fuchs et al., 2021; Andersson, 2021). Health programs that specifically focus on peer support highlight the essential role of social networks in the health of individuals with migration backgrounds. Studies like those by Marchi et al. (2024) show that such programs can significantly improve both the mental and physical health of migrants, emphasizing the importance of integrating social support into healthcare initiatives.

Finally, we investigate how living conditions influence health outcomes, particularly among migrants (H6). Research has shown that the living environment is one of the most critical factors related to health (Bourgois et al., 2017). This not only includes health-damaging factors within the living environment but also includes satisfaction with housing and a sense of belonging (Braveman et al., 2011). Studies from the United States highlight how migration-related residential segregation is embedded in structural patterns that systematically disadvantages migrants (Holt-Lunstad, 2022; Braveman et al., 2018). Precarious housing conditions significantly increase the risk of chronic diseases (Marmot et al., 2012). These conditions not only affect physical health but also contribute to psychological stress and insecurity, which are particularly pronounced during the period shortly after migration. In this phase, when temporary accommodations are often necessary, the risk of developing mental health disorders increases significantly (Mangrio and Zdravkovic, 2018). This is primarily driven by factors such as overcrowded living spaces, lack of privacy, and inadequate sanitary facilities (Ruiz-Tagle and Urria, 2022).

Previous research on migrant health has predominantly focused on factors such as migration status or health-related quality of life. However, this approach overlooks the complex interplay of social mechanisms that shape health outcomes in migrant households. Grounded in Bronfenbrenner's Ecological Systems Theory (Bronfenbrenner, 2000), our study takes a comprehensive approach to advance migration-health research by addressing critical gaps in the existing literature. We move beyond the static treatment of cultural factors by analyzing how family values and relationships dynamically influence health outcomes across migrant generations. This perspective highlights the evolving nature of health determinants within migrant households. Additionally, our focus on household-level social support systems uncovers specific mechanisms within families that shape health behaviors and outcomes, offering insights beyond broad integration indicators. By integrating a comprehensive health index with specific disease patterns, our approach captures the multifaceted health burdens of migrant families more accurately than conventional methods. Moreover, examining the intergenerational transmission of health-related practices reveals how cultural expectations and family dynamics shape long-term health outcomes in migrant communities. Together, these contributions deepen our understanding of the complex social mechanisms influencing migrant health and provide a robust foundation for targeted research and policy interventions.

Based on the theoretical framework and identified research gaps, we test the following hypotheses:

H1 Health outcomes differ between individuals with and without migration background (first/second generation).

H1a The relationship between migration background and health outcomes is moderated by socioeconomic status.

H2 The association between traditional and modern family values and health outcomes differs by migration background.

H3 The relationship between life satisfaction and health outcomes varies by migration background.

H4 The association between parent relationships and health outcomes differs by migration background.

H5 The relationship between social support networks and health outcomes varies by migration background.

H6 The association between living conditions and health outcomes differs by migration background.

## 2 Methods

### 2.1 Data

This study relies on data from the Family Demographic Panel (FReDA), specifically data release v.3.0.0 (DOI: 10.4232/1.14080), as published by Bujard et al. (2023) The Family Demographic Panel is a representative study conducted throughout Germany; surveying individuals aged 18 to 49. The content of the surveys encompasses a comprehensive range of topics pertaining to the lives of families and couples. For a detailed overview of the FReDA panel, please refer to Schneider et al. (2021). This analysis is based on data from the first three subwaves (W1R, W1A, W1B) of the study, comprising a total of 37,815 participants. The sample included all participants with complete data on the primary variables migration status and health (*n* = 8939).

#### 2.1.1 Missing data analysis

Given the substantial amount of missing data in the migration and health variables, a complete case analysis was considered the most robust and transparent approach to maintain the integrity of these variables. The analysis was carefully based on the most consistent and reliable datasets to minimize potential biases in the results.

In addition to the primary variables, for which a complete case analysis was performed, the method of multiple imputation by chained equations (MICE) was applied due to the significant amount of missing data across the dataset (42 % – 77 %). Initially, a predictor matrix was created to define the relationships between variables for the imputation process. The imputation was conducted using the R package “mice” (Van Buuren and Groothuis-Oudshoorn, 2011) applying the Predictive Mean Matching (PMM) method across 20 imputed datasets with a maximum of 50 iterations for each imputation. After the imputation process, the first imputed dataset was extracted and merged with the original dataset using a unique identifier.

#### 2.1.2 Measures and operationalization

This selection of variables was theoretically informed by Bronfenbrenner’s ecological systems theory (Bronfenbrenner, 2000). This framework emphasizes the multilevel nature of individual development and well-being, shaped by interactions across the micro-, meso‑, and macro-levels of the social environment. Our operationalization reflects this logic: micro-level factors (e.g., age, gender, education, loneliness), meso‑level factors (e.g., social support, family values, partnership status, parental relationship), and macro-level influences (e.g., migration status, language background, living conditions) were all included to capture the broad contextual embeddedness of health outcomes. While aspects of the exosystem, such as structural living conditions or indirect effects of household dynamics, are considered, temporal dimensions emphasized in the chronosystem could not be addressed due to the cross-sectional nature of the data (Reifsnider et al., 2005). This multi-layered approach allows us to account for both individual and structural determinants of migrant health.

The analysis includes several socio-demographic characteristics: household income, education level, age, gender, and marital status. We also include psychosocial variables that are closely linked to health outcomes, such as loneliness, life satisfaction, and general well-being. The study also examines social support networks and living conditions as two broader dimensions of the social context. Social support networks include structural and qualitative aspects of parent-child relationships, characteristics of personal support networks, frequency and quality of social contacts. Living conditions include housing size and quality, household composition, health limitations of household members, housing satisfaction and recent degree of urbanization. A detailed overview of all variables, their operationalization, and the specific models in which they were included is provided in the supplementary material (Table S1).

##### 2.1.2.1 Migration status

The migration status was categorized into four groups: (1) No migration background: individuals who were born in Germany, whose parents were both born in Germany, and who hold German citizenship. (2) Second generation: individuals who were born in Germany but have at least one parent born abroad. (3) First generation with German citizenship: individuals who were born abroad and hold German citizenship. And (4) First generation without German citizenship: individuals who were born abroad and do not hold German citizenship (Schenk et al., 2007; Destatis, 2024).

Additionally, the linguistic-cultural background was included. This was divided into: (1) no migration background, and the spoken language is German; (2) individuals with a migration background (first or second generation) who speak German; and (3) individuals with a migration background (first or second generation) who speak a language other than German (Gockeln, 2024).

##### 2.1.2.2 Health status

The health status includes both objective and subjective health dimensions: The objective health status was determined based on the duration of illness, the presence of specific diseases, acute care needs, and limitations in daily activities. The resulting severity index reflects the extent of the health burden, with a higher value indicating a more serious health impairment (including the specific severe illness, a caregiving status, the limitations in daily life, and how long these have already existed).

The subjective health status captures the individual’s perception and assessment of their own health and daily life burden with higher values indicating a more severe health impairment.

##### 2.1.2.3 Traditional and modern family values

To incorporate family values into the model, a latent construct was created encompassing three dimensions: (1) Traditional and modern values regarding family, (2) Traditional and modern values regarding parenthood, and (3) Traditional and modern values regarding the role of mothers. Partnership values include traditional items measuring attitudes towards marriage as a lifelong commitment and women's primary role in family care, while modern items assess the acceptance of unmarried cohabitation and divorce. Parenthood values were measured through traditional items focusing on children as necessity for women's life fulfillment, and modern items examining children as necessity for men's life fulfillment. Maternal role values were captured through traditional items assessing beliefs about young children suffering from maternal employment and women's primary role in family care, while modern items measured attitudes towards the quality of mother-child relationships independent of maternal employment. The model was calculated using complete cases. The fit indices (CFI = 0.994, TLI = 0.977, RMSEA = 0.055, SRMR = 0.019) indicated a good model fit. Factor scores for each dimension were subsequently extracted and added to the main dataset for inclusion in further analyses.

##### 2.1.2.4 Parental relationship

To account for the parental relationship in the model, the dimensions of emotional closeness, frequency of contact per week, perception of a happy childhood, and relationship satisfaction were analyzed separately for mother and father. Initial modelling of both relationships in a single construct showed only satisfactory fit indices (CFI = 0.865, TLI = 0.764, RMSEA = 0.208, SRMR = 0.065). Subsequently, the parental relationship was divided into two separate constructs: mother relationship and father relationship. This division led to a significant improvement in model fit, with very good fit indices for the mother relationship (CFI = 0.993, TLI = 0.978, RMSEA = 0.078, SRMR = 0.019) and the father relationship (CFI = 1.000, TLI = 1.000, RMSEA = 0.008, SRMR = 0.003). The factor scores of the separate parental relationships were then extracted and added to the main dataset to be considered in further models. The R package ‘lavaan’ (Rosseel et al., 2017) was used for both structural analyses.

### 2.2 Statistical methods

The visualizations created using the R packages "ggplot2″ (Wickham et al., 2016) "grid" (R Core Team, 2024), "reshape2″ (Wickham, 2007) "gridExtra" (Auguie, 2017). "tidyr" (Wickham et al., 2024) and "dplyr" (Wickham et al., 2023), provide the foundation for the subsequent multivariate analyses. To examine the effects of migration status on subjective and objective health status, we used multivariate multiple regression models with R packages “lme4” (Bates et al., 2015) and “Matrix” (Bates et al., 2024). The approach allows for the simultaneous analysis of two dependent variables (health severity index and general health status) while controlling for their potential correlation. Six sequential models were specified (see Table S2).

Model 1 (Base Model): Examines the main effects of migration status and household language on health outcomes, controlling for age, gender, education, and household income. An extended version includes specific illnesses and their interactions with migration status.

Model 2 (Family Values): Incorporates traditional and modern attitudes toward marriage, parenthood, and gender roles, including interactions with language proficiency.

Model 3 (Well-being): Adds measures of life satisfaction and happiness, including interactions with household income and language proficiency.

Model 4 (Parent Relations): Examines the impact of parent-child relationships through maternal and paternal relationship scores and intergenerational values.

Model 5 (Social Support): Focuses on social support networks and loneliness, controlling for marital status and household composition.

Model 6 (Living Environment): Analyses the influence of housing satisfaction, urbanisation degree, and household structure.

All models were estimated using ordinary least squares regression, with separate coefficients calculated for each dependent variable. To assess the robustness of our findings, we estimated six different model specifications (Model 1–6), each introducing thematic blocks of predictors (e.g., health conditions, family values, social support, parent relationships, and housing conditions) step by step. This sequential approach allows for the evaluation of the stability of migration-related effects across increasing model complexity. Furthermore, both standardized and unstandardized versions of the dependent variables (severity index and general health status) were tested to assess sensitivity to scale transformation. The consistency of effect directions and significance levels across specifications supports the robustness of the key findings.

## 3 Results

We present our findings in two stages. First, we conduct graphical analyses to illustrate key patterns related to our hypotheses and provide preliminary insights into the relationships between migration, social factors and health outcomes. These visualisations identify underlying patterns in the data and serve as a foundation for the statistical analyses that follow. Then we present results from multivariate regression models that test these relationships while controlling for multiple variables.

### 3.1 Graphical overview of main relationships

The following graphs depict the main relationships in our data and provide context for the subsequent multivariate regression analyses. Corresponding descriptive data can be found in the appendix (Table S1).

**Fig. 1:**
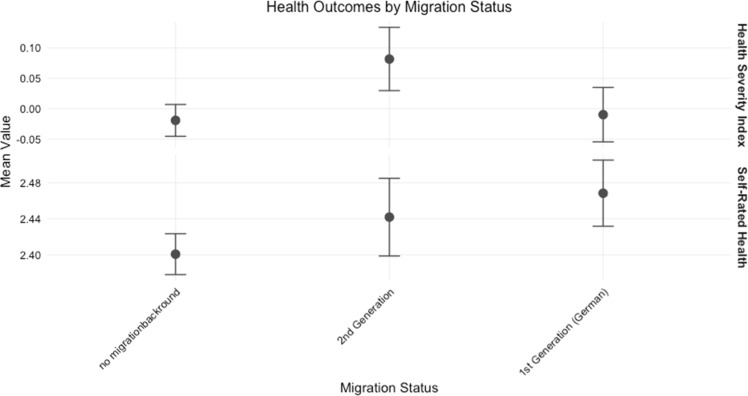
Distribution of general health status by migration background, highlighting disparities aligned with Hypothesis 1. The graph shows mean values and confidence intervals for Health Severity Index and Self-Rated Health across migration status groups. For both measures, higher values indicate more health impairments.

Fig. 1 illustrates health disparities across migration backgrounds, testing our first hypothesis. The Health Severity Index shows notably higher values for second-generation migrants compared to both individuals without migration background and first-generation migrants. In contrast, Self-Rated Health follows a different pattern: both migrant groups report slightly worse subjective health than individuals without migration background. This discrepancy between objective health measures and subjective health perception is particularly pronounced for second-generation migrants.

**Fig. 2:**
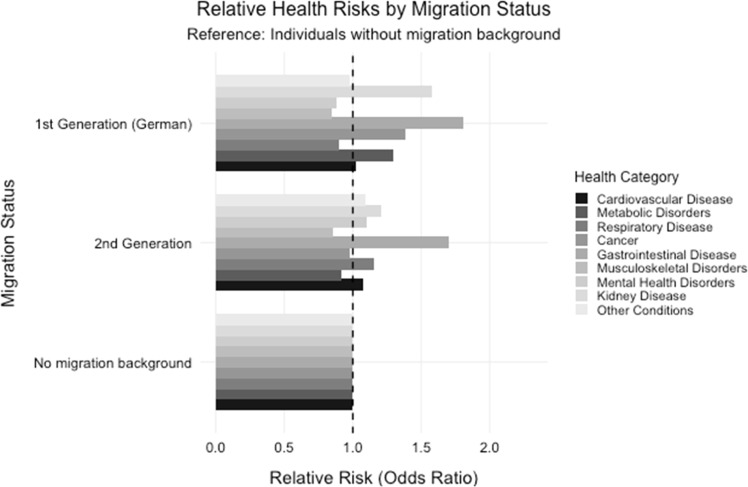
Prevalence of health issues across different migration backgrounds aligned with Hypothesis 1.

Fig. 2 shows odds ratios for various health conditions across migration status groups, with individuals without migration background as reference group (OR = 1.0). The analysis reveals distinct patterns of health risks. Gastrointestinal diseases show elevated risks in both migration groups. Mental health issues are particularly pronounced in the second generation, while kidney disease shows notably higher risk in the first generation. These findings indicate systematic differences in health risks between migration groups, with specific conditions showing varying patterns of disparity.

**Fig. 3:**
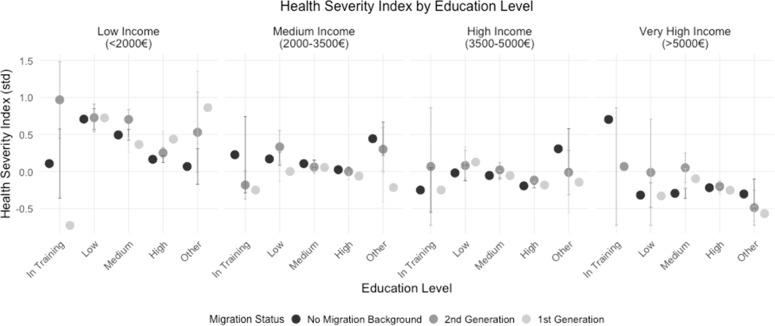
Health status and its relation to migration background, education, and income aligned with Hypothesis 1b.

Fig. 3 shows the Health Severity Index (where higher values indicate poorer health) across different levels of education, separated by income categories: Low Income (<2000€), Medium Income (2000–3500€), High Income (3500–5000€), and Very High Income (>5000€). The x-axis represents different education levels (In Training, Low, Medium, High, and Other), while the y-axis displays the standardized Health Severity Index values.

The figure shows that, overall, the Health Severity Index tends to decrease as increasing income levels across all education levels, suggesting an association between higher income and better health outcomes. Additionally, within each income category, the level of education appears to be linked to health severity: individuals in "In Training" or with "Low" education levels generally have a higher Health Severity Index, indicating poorer health outcomes, especially in the lower income brackets.

In the lower income category, individuals from the 1st generation individuals appear to have slightly higher health severity scores compared to other groups across several education levels, indicating potential health inequalities for this group. Conversely, in the higher income groups, these differences are less pronounced, suggesting that higher income may mitigate some of the health risks associated with migrant status.

In summary, Fig. 3 highlights a complex interaction between income, education, and migration status on health outcomes, with lower income and education levels generally linked to poorer health, and evidence of health disparities for 1st generation migrants, particularly in lower income brackets.

**Fig. 4:**
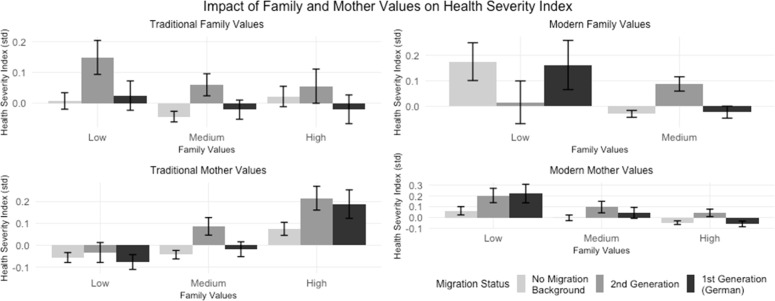
The impact of migration background on attitudes toward marriage, family, and health aligned with Hypothesis 2.

Fig. 4 shows how different dimensions of family values, as defined in the methods section, relate to health outcomes. The analysis distinguishes between traditional and modern orientations, which represent distinct rather than opposite constructs. Traditional family values assess attitudes towards marriage as lifelong commitment and women's family role, while modern family values capture acceptance of unmarried cohabitation and divorce. Traditional mother values reflect beliefs about maternal employment's impact on young children, while modern mother values focus on mother-child relationships in context of maternal employment. The health severity index shows distinct patterns across these value dimensions. For individuals with traditional family values, the Health Severity Index appears to vary across migration status groups. In particular, individuals from the 2nd generation with low traditional family values tend to show poorer health outcomes, while higher traditional values are associated with better health, especially for 1st generation individuals. In the case of modern family values, high values are not well represented in the sample. A medium level of modern family values appears to be associated with better health outcomes, except in the 2nd generation group, where a medium level is linked to poorer health. Low traditional mother values are generally associated with better health outcomes across all groups, while high traditional mother values tend to correlate with poorer health for all groups. On the other hand, low levels of modern mother values appear to be associated with poorer health, while high modern mother values are linked to better health outcomes. In summary**,** Fig. 4 suggests that higher traditional family values seem to be beneficial for health, particularly for the 1st generation group, while higher modern mother values associate with better health outcomes across all groups. These patterns indicate that cultural values related to family and motherhood may influence health disparities based on migration background.

**Fig. 5:**
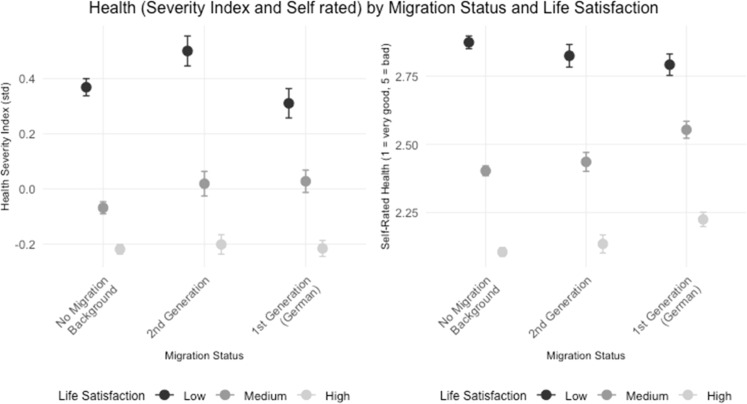
Life satisfaction, happiness, and their link to health status among different migration backgrounds aligned with Hypothesis 3.

Fig. 5 shows the relationship between health (Health Severity Index and Self-Rated Health, where higher values indicate poorer health) and life satisfaction, categorized into three levels (Low, Medium, and High), across different migration groups. Generally, individuals with higher life satisfaction levels exhibit lower Health Severity Index values and better self-rated health, indicating improved health outcomes. Across all migration groups, those with low life satisfaction tend to have higher Health Severity Index values, suggesting poorer health outcomes. This trend is especially pronounced in the 2nd generation and 1st generation groups, where individuals with low life satisfaction show significantly worse health outcomes on the severity index. This effect appears less prominent in self-rated health, where individuals without a migration background show the greatest variance across life satisfaction categories. This suggests that, while life satisfaction consistently impacts health severity across groups, it influences subjective health outcomes most strongly for those without a migration background.

**Fig. 6:**
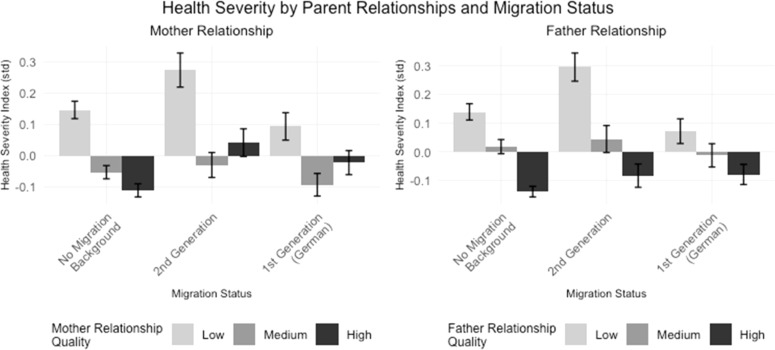
The influence of parental relationships and intergenerational values on health in migration contexts aligned with Hypothesis 4.

Fig. 6 shows the relationship between the Health Severity Index and the quality of relationships with parents across different migration status groups. The figure is divided into two panels, representing the relationship with the mother (left) and the father (right). A good relationship quality with the mother is particularly associated with better health in the no migration background group, while a poorer relationship quality seems to be associated with worse health outcomes, especially in the 1st generation group. Even a medium-quality relationship with the mother seems to correlate with better health across all groups. A high-quality relationship with the father is associated with better health outcomes across all groups, with this effect being most pronounced in the no migration background group, while a lower-quality relationships with the father is linked to poorer health outcomes, particularly in the 2nd generation group. In summary Fig. 6 suggests that the quality of relationships with both parents is associated with health outcomes across migration groups. Higher quality relationships with both mothers and fathers tend to correlate with lower health severity (better health), particulary in the 2nd generation group, where poor relationships with parents are linked to the highest Health Severity Index values. This pattern suggest that parental relationships may play a important role in health inequalities, particularly for individuals in the 2nd generation migrant group.

**Fig. 7:**
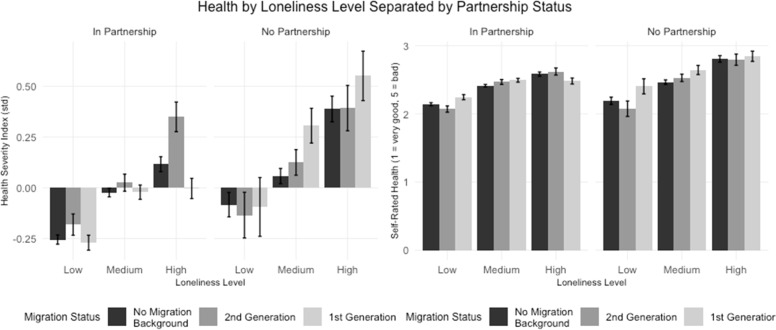
The role of social support in health status variations aligned with Hypothesis 5 ‘.

Fig. 7 shows the relationship between health and levels of loneliness, separated by partnership status across migration status groups. The figure comprises two panels: health severity index (left) and self-rated health (right), with higher values indicating worse health in both measures.

Among those "In Partnership," individuals with lower loneliness levels show lower Health Severity Index values across all migration groups, suggesting better health outcomes. As loneliness levels increase, the Health Severity Index also increases, indicating a correlation between higher loneliness and poorer health. This trend is more pronounced among those "Not in Partnership," where high loneliness is associated with the highest Health Severity Index values, especially in the no migration background group and the 1st generation group.

Similar to the Health Severity Index, lower loneliness levels are associated with better self-rated health outcomes (lower values) across all groups. For those "Not in Partnership," self-rated health declines significantly with increasing loneliness, with individuals experiencing high loneliness reporting the poorest self-rated health. This effect is most noticeable in the no migration background group, followed by the 1st generation group.

In summary Fig. 7 underscores the association between loneliness and poorer health outcomes, with higher loneliness levels correlating with increased health severity and poorer self-rated health across all migration groups. Although loneliness is generally less pronounced among individuals in partnerships, high loneliness levels still appear within this group, with considerable variation in health outcomes across different migration backgrounds.

**Fig. 8:**
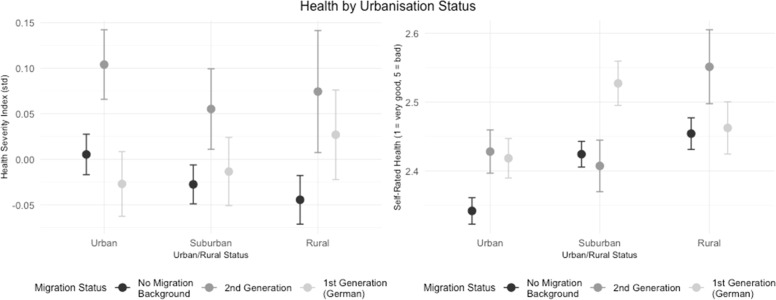
The effect of living conditions on health, with a focus on migration background aligned with Hypothesis 6.

Fig. 8 shows the relationship between health and urbanization status (Urban, Suburban, and Rural) across migration backgrounds. The figure presents both the Health Severity Index (left) and self-rated health (right), where higher values indicate poorer health.

For individuals with no migration background, the Health Severity Index tends to decrease in rural areas, indicating better health outcomes in rural areas compared to urban areas. In contrast, for the 2nd generation and 1st generation groups, the Health Severity Index increases in rural areas, suggesting poorer health outcomes in these areas.

For the 2nd generation, the Health Severity Index is highest in urban areas, suggesting that urban living is associated with poorer health outcomes for this group.

Self-Rated Health presents a different pattern compared to the Health Severity Index. For individuals without a migration background, self-rated health actually worsens in rural areas, suggesting poorer subjective health in these settings. Across all groups, rural areas tend to be associated with poorer self-rated health, while urban and suburban areas show relatively better self-rated health.

In summary Fig. 8 contrast the patterns in health outcomes by urbanisation status, with differences between the Health Severity Index and self-rated health. While the Health Severity Index suggests better health for the non-migrant group in rural areas, the Self-Rated Health measure suggest worse subjective health in rural areas for all groups. For the 2nd generation, both health measures point to the poorest outcomes in rural areas, highlighting potential challenges in these settings. These findings suggest that rural areas may pose particular health risks, especially for migrants. They also emphasize the need to use multiple health measures to fully understand health differences across different levels of urbanization and migration backgrounds.

The initial stage of our analysis involves visualizing main patterns to explore the relationships between migration status, social factors, and health outcomes. These visualizations reveal essential trends and differences within the data, offering preliminary insights that align with our hypotheses. However, visualizations alone are limited in their ability to account for multiple interacting factors that may influence health. To rigorously test the observed relationships while controlling for potential confounding variables, we proceed with multivariate regression models. These models allow us to assess the strength and direction of each relationship independently, providing a more comprehensive understanding of the factors contributing to health disparities across migration backgrounds.

### 3.2 Multivariate multiple regression

Model 1 starts with basic predictors and explains a modest portion of the variance. Significant effects were found for migration status and cultural migration influences (migration language). Additionally, there were significant effects for the control variables age, sex, schooling, and income.

The variance explained by this model was 3.76 % for Health Severity Index and 5.30 % for general health. These results show strong associations with both health severity and general health outcomes, establishing demographic and socioeconomic factors as important in explaining health disparities.

Model 1a further includes specific health conditions to explain the variance in the Health Severity Index and general health outcomes. Significant effects are observed for both migration variables (migration status and migration language) on both health measures, as well as for all control variables (age, sex, education, and income). Cardiovascular disease, metabolic disorders, respiratory disease, cancer, mental health conditions, and kidney disease are all highly significant predictors for both the Health Severity Index and general health, emphasizing their strong impact on health outcomes. Gastrointestinal disorders are marginally significant for general health, indicating a less pronounced but still relevant effect, while other miscellaneous health conditions are highly significant for both health measures, showing that various additional health issues contribute to health disparities. None of the interaction terms between migration language and specific health conditions are statistically significant, suggesting that the effects of health conditions on health outcomes do not vary significantly by migration language. The model explains 30 % of the variance in the Health Severity Index and 13 % in subjective general health. These results suggest that health disparities are strongly influenced by the presence of specific health conditions, in addition to demographic and socioeconomic factors.

**Table 1:** Results from multivariate regression analyses predicting objective (severity index) and subjective (self-rated) health outcomes.

**Parameter**	**Severity Index β** *N* = 8039	**Subjective Health β** *N* = 8039
**Model 1: Base Effects**	R² = 0.037	R² = 0.051
Migration Background	−0.062 (0.028)*	−0.071 (0.028)*
Household Language Use	0.102 (0.040)*	0.129 (0.040)⁎⁎
Age	0.004 (0.001)⁎⁎⁎	0.015 (0.001)⁎⁎⁎
Sex	0.258 (0.021)⁎⁎⁎	0.162 (0.021)⁎⁎⁎
Education	−0.062 (0.006)⁎⁎⁎	−0.087 (0.006)⁎⁎⁎
Household Income	−2.12e-5 (2.77e-6)⁎⁎⁎	−1.67e-5 (2.75e-6)⁎⁎⁎
Marital Status	−0.089 (0.021)⁎⁎⁎	−0.059 (0.021)⁎⁎
**Model 1a: Health Conditions**	R² = 0.302	R² = 0.135
Migration Background	−0.082 (0.033)*	−0.077 (0.036)*
Household Language Use	0.077 (0.035)*	0.100 (0.039)*
Cardiovascular Disease	0.728 (0.056)⁎⁎⁎	0.259 (0.062)⁎⁎⁎
Metabolic Disorders	0.091 (0.026)⁎⁎⁎	0.258 (0.029)⁎⁎⁎
Respiratory Disease	0.279 (0.028)⁎⁎⁎	0.203 (0.032)⁎⁎⁎
Cancer	0.770 (0.059)⁎⁎⁎	0.344 (0.065)⁎⁎⁎
Gastrointestinal Disease	0.048 (0.080)	0.259 (0.089)⁎⁎
Musculoskeletal Disorders	0.140 (0.024)⁎⁎⁎	−0.022 (0.027)
Psychological Disorders	1.156 (0.028)⁎⁎⁎	0.564 (0.031)⁎⁎⁎
Kidney Disease	0.696 (0.098)⁎⁎⁎	0.489 (0.109)⁎⁎⁎
Other Conditions	0.461 (0.026)⁎⁎⁎	0.430 (0.029)⁎⁎⁎
**Model 2: Cultural Values**	R² = 0.053	R² = 0.065
Migration Background	−0.013 (0.029)	−0.012 (0.029)
Household Language Use	0.049 (0.042)	0.063 (0.042)
Traditional Family Values	−0.794 (0.175)⁎⁎⁎	−0.461 (0.174)⁎⁎
Modern Family Values	−1.618 (0.347)⁎⁎⁎	−0.851 (0.345)*
Traditional Mother Role	0.421 (0.066)⁎⁎⁎	0.360 (0.066)⁎⁎⁎
Modern Mother Role	0.285 (0.056)⁎⁎⁎	0.212 (0.055)⁎⁎⁎
**Model 3: Well-being**	R² = 0.138	R² = 0.225
Migration Background	0.067 (0.058)	−0.116 (0.055)*
Household Language Use	0.026 (0.039)	0.038 (0.037)
Life Satisfaction	−0.046 (0.009)⁎⁎⁎	−0.096 (0.009)⁎⁎⁎
Happiness	−0.126 (0.008)⁎⁎⁎	−0.147 (0.007)⁎⁎⁎
Income × Life Satisfaction	n.s.	−3.36e-6 (1.35e-6)*
**Model 4: Family Relations**	R² =0.058/	R² = 0.078
Migration Background	−0.052 (0.032)	−0.086 (0.033)⁎⁎
Household Language Use	0.080 (0.046)	0.124 (0.047)⁎⁎
Father Relationship	−0.030 (0.017)+	−0.070 (0.017)⁎⁎⁎
Mother Relationship	−0.120 (0.019)⁎⁎⁎	−0.144 (0.019)⁎⁎⁎
Mother in Household	−0.174 (0.066)⁎⁎	−0.215 (0.067)⁎⁎
Contact Frequency Mother	0.033 (0.008)⁎⁎⁎	0.035 (0.008)⁎⁎⁎
Language × Mother Relationship	0.063 (0.025)*	0.055 (0.025)*
Language × Father Relationship	−0.040 (0.021)+	n.s.
**Model 5: Social Integration**	R² = 0.060	R² = 0.088
Migration Background	−0.055* (0.028)	−0.059 (0.023)
Household Language Use	0.035 (0.086)	0.186 (0.072)*
Loneliness	0.033 (0.004)⁎⁎⁎	0.051 (0.004)⁎⁎⁎
Informal Support Network	−0.031 (0.009)⁎⁎⁎	−0.035 (0.009)⁎⁎⁎
Currently partnered	0.157 (0.026)⁎⁎⁎	0.141 (0.022)⁎⁎⁎
Migration Status × Loneliness	n.s.	−0.010 (0.004)⁎⁎
**Model 6: Living Conditions**	R² = 0.056	R² = 0.088
Migration Background	−0.102 (0.042)*	−0.035 (0.042)
Household Language Use	0.074 (0.042)	0.089 (0.041)*
Housing Satisfaction	−0.061 (0.007)⁎⁎⁎	−0.092 (0.007)⁎⁎⁎
Degree of Urbanization	−0.034 (0.017)*	0.050 (0.017)⁎⁎
Health Restrictions Household	2.75e-5 (1.14e-5)*	2.80e-5 (1.12e-5)*
Household Size	−0.040 (0.010)⁎⁎⁎	−0.026 (0.010)⁎⁎
Migration Status × Urban-Rural	0.030 (0.016)+	n.s.

Note:, n.s. = not significant. Standard errors in parentheses. All continuous variables are z-standardized. Spoken Language categorizes individuals with migration background into German-speaking and non-German speaking in everyday communication. All models control for age, sex, education, household income, and marital status, Coefficients for these control variables are not displayed for clarity but are available upon request.

⁎ *p* < 0.05,

⁎⁎ *p* < 0.01,.

⁎⁎⁎ *p* < 0.001,.

+ *p* < 0.10.

Model 2 introduces family and value-oriented variables, which show significant associations with health outcomes. Traditional and modern values, particularly those related to family and motherhood, appear to be important factors in health, with higher family values linked to better health outcomes. Significant migration effects are observed for subjective health but not for the objective health measure (Health Severity Index). Additionally, control variables (age, sex, education, income) and marital status show significant effects. Significant main effects are also found for family values, both traditional and modern, suggesting that family values are meaningful contributors to health. However, no significant interaction effects are found between migration status and family values, indicating that the relationship between family values and health does not differ significantly by migration background. The model explains 5 % of the variance in the Health Severity Index and 6 % in subjective health.

Model 3 introduces variables related to life satisfaction and happiness, adding depth to our understanding of the determinants of individual well-being on health outcomes. No significant effects are found for migration status or migration language on either the Health Severity Index or general health, suggesting that migration-related variables do not provide additional explanatory power in this model. Age, sex, education, and income remain highly significant predictors for both health outcomes, consistent with previous models, highlighting the strong and persistent influence of demographic and socioeconomic factors on health.

Both measures of life satisfaction are highly significant for both health outcomes (*p* < 0.001), indicating that higher life satisfaction is associated with lower health severity and better general health. This underscores the importance of subjective well-being as a key factor in health outcomes. No significant interaction effects are found between migration language and life satisfaction measures, suggesting that the relationship between life satisfaction and health does not vary by migration background. The model explains 13 % of the variance in the Health Severity Index and 22 % in subjective general health. These results highlight life satisfaction as a significant predictor of health, with stronger explanatory power for subjective health. The findings emphasize the importance of subjective well-being factors, suggesting that life satisfaction plays a crucial role in shaping health outcomes.

Model 4 introduces variables related to the quality of parental relationships (mother and father), as well as additional family relationship scores, to assess their influence on health outcomes. Migration status is not significant for the Health Severity Index but is significant for general health (*p* < 0.01), suggesting that migration background continues to play a role in subjective health but not necessarily in objective health severity. Similarly, migration language is not significant for the Health Severity Index but is significant for general health (*p* < 0.01), indicating a cultural influence on self-rated health outcomes. Age, sex, education, and household income remain highly significant for both health outcomes, reinforcing their consistent importance across models. In terms of family relationships, both mother and father relationship quality scores are significant for both Health Severity Index and general health, showing that a positive relationship with parents is associated with better health outcomes. Structural relationship factors, such as whether parents live in the household or the frequency of contact, are also significant, but only for mother-related variables. Interestingly, frequent contact with the mother is associated with poorer health outcomes, suggesting that the nature and dynamics of mother-child relationships may impact health. There are significant interaction effects between migration language and mother relationship quality for both Health Severity Index and general health (*p* < 0.05), indicating that the impact of the mother relationship on health varies by cultural context. Additionally, a significant interaction effect is observed between migration language and father relationship quality for the Health Severity Index (*p* < 0.05), but not for general health, suggesting that migration background may influence the relationship between father relationships and health severity. The model explains 5.8 % of the variance in the Health Severity Index and 8 % in subjective general health. These results highlight the critical role of family relationships, particularly with parents, in shaping health outcomes. The significant interactions between migration language and parental relationship scores suggest that the influence of family relationships on health may differ across cultural contexts, providing valuable insights into the role of social and cultural factors in health disparities.

Model 5 introduces variables related to social support. In this model, migration variables are significant, with cultural background being significant for subjective health and marginally significant for the Health Severity Index, suggesting that cultural factors may play a role in self- subjective health but not in objective health severity. The added variables, loneliness and social support network, are both highly significant for the Health Severity Index and general health (*p* < 0.001). Higher loneliness is associated with poorer health outcomes, while having someone to discuss personal matters with (a measure of social support) is linked to better health. Additionally, being in a partnership is associated with better health outcomes, suggesting a protective effect for partnered individuals on objective health measures. A significant interaction effect is observed between migration background and loneliness, indicating that migration background may affect how loneliness impacts health. The model explains 6 % of the variance in the Health Severity Index and 8.8 % in subjective general health. These results underscore the significant impact of loneliness and social support on health, with loneliness contributing negatively to health outcomes and social support acting as a protective factor. This model suggests that social connectedness and support are crucial for health, especially in the context of migration, where isolation may be a particular risk factor.

Model 6 includes additional environmental and contextual factors, such as urbanization and household restrictions, to further explore their impact on health outcomes. Migration status remains significant for both the Health Severity Index and general health, with a slight negative effect, suggesting that migration background continues to contribute to health disparities. Migration language is significant for general health, indicating a cultural influence on subjective health outcomes. Age, sex, education, and household income continue to be highly significant predictors for both health outcomes, reinforcing their persistent importance. Urbanization shows a significant effect on general health, with rural or suburban environments potentially impacting health differently compared to urban settings. Household limitations are also significant for both health outcomes, indicating that restrictions or challenges within the household negatively affect health. Housing satisfaction is highly significant for both health outcomes, showing that higher satisfaction with one’s living situation is associated with better health, underscoring the importance of housing as a factor in well-being. The model also includes interaction terms to examine how migration background interacts with urbanization and household limitations. However, these interactions are not significant, suggesting that the effects of environmental and household factors on health are consistent across migration backgrounds. The results suggest that housing satisfaction, urbanization, and household challenges are important predictors of health, especially in the context of migration. The model explains 5.6 % of the variance in the Health Severity Index and 9 % in subjective general health.

In summary, the findings can be synthesised as follows. Hypothesis 1 can be partially confirmed. There are significant differences in health status between migrants and non-migrants, which also vary in connection with socio-economic factors. While migration influences on the objective health burden are less strong, they still have a significant influence on subjective health, which indicates cultural differences in health assessment.

Hypothesis 2 is also partially confirmed. Family values show significant correlations with health outcomes, especially among migrants. This emphasises the role of cultural values as an influencing factor on health. Hypothesis 3 is fully confirmed, as high life satisfaction and happiness are strongly associated with better health outcomes. These results show that subjective well-being is a key predictor of health, regardless of migration status.

Hypothesis 4 can also be partially confirmed. The quality of relationships with parents has a significant impact on health, especially in migrant families. This indicates that strong family ties can play an important role in the health process.

Hypothesis 5 is fully confirmed, as social support has a protective effect on health, while loneliness is significantly negatively associated with health outcomes. This emphasises the importance of social networks and shows that loneliness is a significant risk factor for health.

Hypothesis 6 is also confirmed. Housing satisfaction shows strong positive effects on health, emphasising the importance of housing conditions as a key environmental factor for well-being.

Our results provide supportive evidence for the role of migration, family values, life satisfaction, social support and housing conditions as important determinants of health. In the following discussion, we elaborate these aspects and examine in particular the specific challenges and protective factors associated with migration.

## 4 Discussion

### 4.1 The interplay of values, well-being, and health

The results of our analyses reveal a complex picture of the health situation of migrants. This perspective offers a useful framework to better understand health as individual behavior within a multilayered context and highlights the dynamic interactions between individuals and their social, local, and cultural environments (Reifsnider et al., 2005).

Contrary to expectations, we found that the direct effects of migration prove to be weak and diminish further when we control for value orientations and social factors. This suggests that it is not the migration status itself but rather the associated social and cultural factors that are responsible for health disparities. The inclusion of health status (Model 1b: R²=0.302) and particularly life satisfaction (Model 3: R²=0.225) substantially improves model fit, underscoring their explanatory relevance. The strong negative effects of both overall life satisfaction (β=−0.046***) and happiness (β=−0.126***) on health highlight how important psychosocial factors are for health. The effects persist even after controlling for sociodemographic factors and migration status. This relationship extends well beyond traditional biomedical explanatory approaches, which primarily focus on biological and physiological factors of health, and aligns with current research findings on the close interconnection between subjective well-being and health (Diener et al., 2017).

These findings support previous research which highlights subjective well-being as an integral measure for evaluating migration experiences (Hendriks and Bartram, 2019). High life satisfaction is cited as an indicator of successful migration (Paloma et al., 2021). Moreover, life satisfaction could be interpreted not only as an indicator of integration success but also as a significant predictor of return migration behavior (Schiele, 2021). As previous studies have shown, migrants with low life satisfaction are more likely to consider returning to their country of origin. This association could be relevant in the present study. Individuals with low life satisfaction, especially in combination with poor health, may be more likely to drop out of the study. This could result in a ceiling effect, where the remaining sample primarily consists of individuals with above-average life satisfaction and better health conditions. Such an effect could bias the results and limit their generalizability to the overall migrant population. It might also be related to the previously mentioned Healthy Migrant Effect, where healthier and more resourceful individuals are initially overrepresented in the migrant population (see limitation section for further discussion).

Another key finding is the unexpectedly strong effect of value orientations on health. Both traditional and modern family values show significant negative correlations with health impairments, with modern value orientations exhibiting the strongest effect (β=−1.618***). These results suggest that it is not the specific content of the values but rather the mere presence of stable value orientations that has a health-promoting effect. Notably, these value effects far outweigh the strength of classical socioeconomic determinants. Findings from other studies suggest that economic and cultural factors are associated but also independently influence health. Particularly regarding children's health, strong cultural effects have been observed, which not only impact immediate health but may also serve as a long-term factor in health inequalities, for instance through family conflicts and the quality of family interactions (Sweeting and West, 1995).

### 4.2 The importance of social support

The effects of value orientations indicate that health is significantly influenced by social and cultural factors. Value orientations do not appear to act in isolation but are closely intertwined with social relationships and support structures (Fadiji and Khumalo, 2023).

This becomes particularly evident when considering the role of social support and loneliness. Model 5 demonstrates that loneliness negatively impacts both objective and subjective health (β_sev_=0.033***), while social support (β_sev_=−0.032) has a protective effect. Particularly noteworthy is the observation that the negative effect of loneliness on health persists even after controlling for partnership status and other structural factors. This finding suggests limitations in using partnership status as a sole indicator of social support in relation to health. The presence of high loneliness within partnerships suggests that simply being in a relationship may not always equate to adequate social support, particularly in the context of health challenges. This complexity highlights the need for a more nuanced understanding of social support, beyond partnership status, to accurately assess its impact on health outcomes.

The importance of social support and loneliness for both mental and physical health outcomes was starkly highlighted during the COVID-19 pandemic. As studies have shown, pandemic-related isolation and social distancing had significant impacts on mental health (Laham et al., 2021). Furthermore, meta-analyses also indicate clear links to overall mortality, with loneliness and social isolation showing direct effects on physical health outcomes such as cardiovascular diseases and cancer (Holt-Lunstad, 2024).

Close family relationships are a key resource for health and health-promoting behaviors (Ferrer et al., 2005). In this study, general social support, and particularly the relationship with parents, especially with the mother emerged as significant protective factors for health. The effects of the parent-child relationship remained stable even when socioeconomic factors were controlled for. While a strong mother-child relationship has a markedly protective effect on health, more frequent contact (measured as the number of days per week) was associated with poorer health outcomes (β=0.033*). This seemingly paradoxical finding warrants further interpretation, as several mechanisms may explain this counterintuitive association.

One possible explanation is reverse causality, where health problems drive increased contact rather than contact affecting health. Individuals with poorer health may seek or require more parental support, increasing contact frequency. Another interpretation relates to caregiving burden, particularly in migrant families where adult children often assume caregiving responsibilities for older family members (Shrestha et al., 2023). Hynek et al. (2023) describes how the intersection of caregiving responsibilities and migration background can result in a "double jeopardy" with amplified risks for both physical and mental health. In addition, migration policies themself can create complex dependency structures within families. As Strasser et al. (2009) argues, family migration regulations may introduce dependencies within the family. Legal regulations can create situations where family members depend on one another to meet residence or financial requirements. These rules can increase stress and limit individual freedom within the family. In such cases, frequent contact may not reflect closeness by choice, but obligation, factors that can strain relationships and harm health.

However, the frequency of contact alone does not fully capture the nature of parent-child dynamics. These differentiated effects of the parent-child relationship highlight the independent significance of family relationships for health. The findings expand on those of Stewart-Brown et al. (2005), who initially examined the influence of early parent-child relationships on later health outcomes. They showed that poor relationship quality with parents during childhood is associated with a higher likelihood of multiple health problems in adulthood (Stewart-Brown et al., 2005). Our analyses demonstrate that the quality of the parent-child relationship retains its independent importance for health into adulthood, even when controlling for gender, social class, and mental health. This underscores the enduring relevance of the parent-child relationship throughout life and suggests that the context and quality of parent-child interactions, rather than mere contact frequency, are crucial for understanding their health implications.

### 4.3 Living conditions as a key factor

The urbanization factor reveals differences in health between urban, rural, and suburban areas. This effect varies for subjective and objective health, indicating that people in rural areas tend to feel subjectively less healthy, even though their objective health is better. This could point to heightened health concerns, possibly due to inadequate medical care. Notably, the positive health effect in rural areas predominantly applies to individuals without a migration background. The nearly significant interaction effect of migration and rural areas indicates that people with a migration background tend to be less healthy. This is particularly significant as it implies a structural disadvantage for migrants in rural regions. This disadvantage shaped by several interconnected factors. On the one hand, the inadequate health infrastructure in rural areas, characterized by longer distances to medical facilities, reduced mobility, fewer specialized medical services, and the lack of voluntary support networks (Rösch et al., 2020). Additionally, the social structure of rural populations which is often marked by less developed intercultural experiences and competencies, plays a role. This leads to limitations in the availability and quality of psychosocial resources that are crucial for health (Glorius et al., 2022).

It is also interesting to note that a higher number of people living in a household appears to correlate with better health. The more people live in a household, the better the health outcomes. This suggests a "protective" effect of larger households. Possible explanations could include social support in daily life and a better distribution of burdens. This is particularly intriguing in the context of the significant variable "Health Restrictions Household". Despite potentially more sick household members, the positive effect of larger households seems to prevail. The findings in the literature vary depending on the type of illness: while positive effects of larger households have been observed in the case of lifestyle-related illnesses (e.g., cardiovascular health due to shared routines or meal preparation), negative impacts on household members are more evident if one or more of them suffer from mental illnesses (Lee et al., 2019; Hughes and Waite, 2002). Furthermore, it remains unclear to what extent the size of the living space might moderate these effects, an aspect that needs further investigation. The bidirectional relationship is particularly noticeable in child health. On the one hand, larger household size can negatively affect children's physical development through resource competition, limited personal space, and higher exposure to illness. On the other hand, the presence of multiple household members, regardless of whether the children are young or adult, can offer protective effects, including emotional support, shared caregiving responsibilities, and enhanced social connections (Ntshebe et al., 2019; Hughes and Waite, 2002; Rattay et al., 2012). Future research should further differentiate the dynamics of these effects between young and adult children to capture the nuances of household interactions.

The results of Model 6 show strong effects of housing satisfaction on both subjective and objective health. The particularly strong effect on subjective health (R² = 8.8 %) highlights the close relationship between housing satisfaction and health perception. Study results on socially disadvantaged groups indicate that individual and internal factors in the construct of housing satisfaction play a central role. These factors are closely linked to life satisfaction, which in turn can be strongly associated with one’s self-assessment of health. In particular, in disadvantaged life situations, subjective perception and the way people deal with housing conditions can significantly influence how their perceived and evaluated their own life and health situation (Tsai et al., 2012). Results from Germany suggest that people with lower social status are not only less satisfied with their housing situation but also exhibit less favorable health behaviors. These findings highlight how social inequalities can affect not only life satisfaction but also health and behavior (Koller et al., 2010).

### 4.4 Migration effects and the persistent influence of demographic and socioeconomic factors

The models consistently demonstrate that factors such as age, gender, education, and income are reliable predictors of health. These fundamental variables also interact with migration status and cultural factors, highlighting that health disparities cannot be solely attributed to migration background. This finding is supported by studies from Germany, which emphasize social inequalities in education and income as central explanatory factors for health disparities (Lampert and Kroll, 2014). The results can be interpreted within the framework of the social gradient hypothesis, which describes the distribution of health inequalities as a universally acting mechanism, regardless of whether they occur in resource-rich or resource-poor contexts (Marmot, 2006). Primary determinants (such as the control variables here) form the basis for health differences. At the same time, the analyses presented here reveal that the combination of these primary effects with psychosocial factors produces complex interactions that require nuanced consideration (Kosteniuk and Dickinson, 2003).

The present models reveal three key effects that will be discussed in more detail. Initially, a significant effect of migration status on health was observed. However, this effect diminishes or disappears in the models when cultural and social factors are taken into account. This suggests that it is not migration status itself that is decisive for health disparities but rather the associated cultural and social conditions. This becomes particularly evident in Model 2, which focuses on value orientations. Values and norms relevant in the migration context influence health more strongly than status itself. Studies confirm that social and cultural factors such as values, family roles, and social networks play a crucial role in health inequalities (Addae, 2020). On the other hand, stronger effects of migration status are observed for both subjective and objective health, with the effects being more pronounced for the subjective health variable. This discrepancy could be explained by culturally driven differences in illness perception and coping strategies. Furthermore, these effects could indicate communicative challenges within the healthcare system. Difficulties in communication with healthcare professionals or the perception of discrimination may amplify the subjective experience of health problems (Marmot, 2005). This shows that in addition to medical indicators psychosocial factors also play a central role (Amoah and Oteng, 2024).

Building on this, the third key effect reveals distinct patterns for the two migration variables: migration status and spoken language. Spoken language represents a combined categorization based on migration background (first or second generation) and whether German or a non-German language is spoken in the household. The analysis shows that individuals from households where a non-German language is predominantly spoken face increased health risks. These findings suggest that linguistic barriers are not merely a communication issue but represent a structural barrier to equal healthcare access. Individuals experiencing these barriers might avoid or delay seeking medical help, misunderstand treatment plans, or fail to adhere to medical instructions, all of which contribute to poorer health outcomes. These observations align with existing research highlighting language as a significant indicator of health risks (Paloma et al., 2021). Studies demonstrate that language barriers can significantly hinder access to healthcare information, limit effective use of healthcare services, and impair communication with medical staff (Al Shamsi et al., 2020). Such barriers can result in delays in care, misdiagnoses, or inadequate treatments, ultimately posing serious health risks (Divi et al., 2007).

### 4.5 Limitations

The present study exhibits methodological particularities that must be considered when interpreting the results. Data collection was conducted in three consecutive subwaves. Although this could theoretically influence the assumption of simultaneity in measurements, several factors support the robustness of the analyses. First, the temporal gap between data collection waves was only a few weeks. Furthermore, the constructs examined, particularly values and norm orientations, exhibit high temporal stability, and additional sensitivity analyses revealed no systematic differences between the subwaves.

The representativeness of the sample may be limited, which could affect the generalizability of the findings. This limitation primarily pertains to the selection of the sample with complete data for the health and migration variables (as outlined in the Methods section) and the underrepresentation of individuals without German citizenship. Our response analysis revealed significant differences in data completeness, with only 22.4 % of first-generation migrants without German citizenship having complete health data, compared to 45.4 % of those without migration background. This systematic pattern likely results in the underrepresentation of more vulnerable migrant groups, particularly recent arrivals, who typically face greater barriers to healthcare access (Hacker et al., 2015). Consequently, the reported health disparities may be underestimated. The health profiles identified in this study therefore primarily reflect the situation of relatively well-integrated migrant populations and should be interpreted with caution when generalizing to more marginalized groups.

To ensure the validity of the findings, a differentiated approach to handling missing data was employed, focusing on both empirical authenticity and methodological robustness. For the primary variables, particularly health and migration status, a Complete Case Analysis was deliberately applied. This approach aimed to reflect actual empirical relationships and avoid distortions caused by statistical imputations. For supplementary analyses and control variables, the Multiple Imputation by Chained Equations (MICE) method was used. This enhanced the efficiency of the estimates while maintaining the comprehensiveness of the analyses.

The parallel application of both strategies not only ensured the robustness of the findings but also safeguarded their authenticity. This methodological approach adhered to the principle of conservative estimation: the objective was to produce fewer, but more reliable and valid results, rather than generating potentially artificial associations through statistical methods. The consistency of findings across both approaches underscores the reliability of the observed relationships.

### 4.6 Conclusion

Our study contributes to a more differentiated understanding of the complex interplay between migration and health by illustrating that health disparities are not primarily driven by migration status itself, but rather by the surrounding social and cultural context in which migration is embedded.

This is particularly evident in the central role of psychosocial factors, especially life satisfaction and subjective happiness. The strong negative effects of these factors on health impairments underscore their significance far beyond traditional biomedical explanatory models. These insights are complemented by the unexpectedly strong impact of value orientations on health. The finding that both traditional and modern family attitudes correlate significantly negatively with health impairments suggests that it is not the specific nature of values but rather the presence of stable value orientations that promotes health.

In this context, the central role of social support systems, particularly family relationships, also gains importance. The quality of the parent-child relationship especially with the mother proves to be an important protective factor for health. This effect remains stable even when controlling for socioeconomic factors. This finding emphasizes the need to recognize family ties and social networks as health-relevant resources and to incorporate them into healthcare provision.

These findings have far-reaching implications for the healthcare of individuals with migration backgrounds. Community-based approaches that take cultural backgrounds and family contexts into account offer promising solutions for improved healthcare. Concrete initiatives could include integrated community health centers that combine preventive care with social services, offering culturally sensitive health education. These centers should also offer support structures for informal caregivers, including counseling and training, to mitigate the health risks associated with high caregiving burden, especially among migrant women (Shrestha et al., 2023). Another promising idea could be the development of family-based health promotion programs that address intergenerational dynamics and strengthen family ties as a resource for health. To be effective, such initiatives should be embedded in local communities and co-created with them, supported by intersectoral collaboration and efforts to reduce structural barriers (Shrestha et al., 2023). In addition to creating new structures, a focus on context sensitivity should be prioritized to address individual living conditions (Powell et al., 2024). A transcultural approach in nursing aligns with efforts to promote structural competence in healthcare, helping professionals address the challenges of diversity in clinical practice (Bourgois et al., 2017).This results highlight the necessity of a transcultural orientation in healthcare, systematically considering cultural differences and integrating them into practice (Fischer, 2021). In summary, our findings demonstrate that it is not migration status itself but the complex social determinants surrounding it that explain health disparities. This insight is highly relevant for both future research and the development of equitable health policies.

**Declaration** of generative AI and AI-assisted technologies in the writing process- During the preparation of this work, the first author used ChatGPT to assist with language suggestions and content clarification. After using this tool, the author reviewed and edited the content as needed and take(s) full responsibility for the content of the publication.

## CRediT authorship contribution statement

**Franziska Reinhardt:** Writing – review & editing, Writing – original draft, Visualization, Software, Methodology, Investigation, Formal analysis, Data curation, Conceptualization. **Imad Maatouk:** Validation, Supervision, Software, Resources, Project administration, Funding acquisition.

## Declaration of competing interest

The authors declare that they have no known competing financial interests or personal relationships that could have appeared to influence the work reported in this paper.

## Appendix Supplementary materials

Image, application 1

Image, application 2
