# Supplementary material for: Beyond individual integration: Family systems, social support networks and living environment as health determinants among migrants in Germany
Source: J Migr Health. 2025 Oct 17;12:100368. doi: 10.1016/j.jmh.2025.100368 (PMC12637097; doi:10.1016/j.jmh.2025.100368)
Supplement: Supplementary file 1 [file mmc1.pdf]

Table S1: Descriptive Statistics by Migration Status

	Sample	Health		Sociodemographic				Well-being		Family			
Group	N	Severity Index	Self-Rated Health	Age	Female	Education	Income	Life Sat.	Happiness	Trad. Values	Mod. Values	Mother Rel.	Father Rel.
No migration background	5,561	1.48 (2.09)	2.40 (0.86)	35.61 (8.82)	54.1	5.80 (1.64)	4.07 (4.21)	6.65 (1.98)	7.10 (1.88)	-0.03 (0.63)	0.01 (0.26)	0.02 (1.00)	0.06 (1.10)
2nd Generation	1,521	1.68 (2.17)	2.43 (0.86)	32.03 (9.49)	58.1	5.76 (1.78)	3.53 (2.61)	6.48 (2.01)	6.84 (2.08)	0.04 (0.71)	-0.02 (0.31)	0.01 (1.05)	-0.12 (1.17)
1st Gen. (German citizenship)	1,857	1.52 (2.07)	2.47 (0.81)	39.92 (6.26)	53.9	5.54 (1.67)	3.82 (3.27)	6.77 (1.91)	7.20 (1.91)	0.06 (0.70)	-0.02 (0.29)	-0.07 (1.05)	-0.10 (1.17)

Note: First-generation migrants without German citizenship are systematically underrepresented in the sample due to response pattern and are therefore not included in this descriptive analysis (see limitations section and methodological paper for details. Values are presented as means with standard deviations in parentheses. Female values are percentages. Health: Severity index ranges from 0 to 12 (higher values indicate more health problems), General health ranges from 1 (very good) to 5 (very poor). Well-being measures range from 0 (lowest) to 10 (highest). Income in thousand Euros; Traditional values, modern values, and parent relationship scores are z-standardized."
