# Supplementary material for: Beyond individual integration: Family systems, social support networks and living environment as health determinants among migrants in Germany
Source: J Migr Health. 2025 Oct 17;12:100368. doi: 10.1016/j.jmh.2025.100368 (PMC12637097; doi:10.1016/j.jmh.2025.100368)
Supplement: Supplementary file 2 [file mmc2.pdf]

Table S2: Overview of Model Structure, Key Variables, and Operationalizations

	Model 1 *Base Effects*	Model 1a *Health Conditions*	Model 2 *Cultural Values*	Model 3 *Well-being*	Model 4 *Family Relations*	Model 5 *Social Integration*	Model 6 *Living Conditions*
**Dependent Variables**	Subjective & Objective Health Status						
**Key Variables**	Migration Background & Household Language Use						
		Diseases (Cardiovascular D., Metabolic D., Respiratory D., Cancer, Gastrointestinal D., Musculoskeletal D., Kidney D., Mental D., Other D.)	Traditional /Modern Family Values<br>Traditional/ Modern Parents Values<br>Traditional/ Modern Mother Values	Life Satisfaction<br>Happiness	Father/ Mother Relationship<br>Mother/ Father living in Household<br>Weekly Contact Frequency (Mother/Father)	Loneliness<br>Informal Support Network<br>Currently partnered	Housing Satisfaction<br>Degree of Urbanization<br>Health Restrictions in Household<br>Household Size
**Interactions**		Migration Background x Diseases	Language spoken at home x family values	Income × Life Satisfaction<br>Migration Background x Life Satisfaction<br>Migration Background x Happiness	Language spoken at home × Mother Relationship<br>Language × Father Relationship	Migration Status × Loneliness	Migration Status × Urban-Rural
**Controls**	Age, Sex, Education, Income, marital status						
**Operationalizations**	Dependent variables: Objective Health Status: Index based on disease severity, caregiving status, activity limitations, and duration of illness (standardized); Subjective Health Status: Self-rated health (standardized).<br>All Models) Self-reported primary language used in everyday life, coded as: (0) no migration background, German spoken; (1) migration background, German spoken; (2) migration background, non-German spoken; Migration Background based on place of birth and citizenship: (0) No migration background, (1) Second generation, (2) First generation with German citizenship, (3) First generation without German citizenship.<br>M1) Self-reported doctor-diagnosed conditions (binary): cardiovascular, metabolic, respiratory, cancer, gastrointestinal, musculoskeletal, kidney, mental health, other.<br>M2) Values - Latent constructs of family values based on agreement with traditional (e.g., marriage permanence, maternal care) and modern (e.g., acceptance of divorce/cohabitation, maternal employment) value items.<br>M3) Life Satisfaction: Rated from 0 (not satisfied) to 10 (completely satisfied). Happiness: Rated from 0 (extremely unhappy) to 10 (extremely happy).<br>M4) Mother/father relationship (latent): Based on emotional closeness, relationship satisfaction, childhood relationship quality, and instrumental support received from mother/father (4 indicators each); Mother/Father Living in Household Binary indicator for whether the mother or father resides in the same household (1 = yes, 0 = no); Contact Frequency Mother/Father Number of days per week the respondent is in contact with their mother or father.<br>M5) Loneliness is a summated scale based on six items (e.g., "I feel isolated"), higher scores indicate greater loneliness; Informal Support Network Number of people the respondent can turn to for help with personal matters; Currently Partnered Binary indicator for being in a relationship (married or cohabiting) at the time of the survey						

Table S2: Overview of Model Structure, Key Variables, and Operationalizations

	**Model 1** *Base Effects*	**Model 1a** *Health Conditions*	**Model 2** *Cultural Values*	**Model 3** *Well-being*	**Model 4** *Family Relations*	**Model 5** *Social Integration*	**Model 6** *Living Conditions*
	M6) Household Size-Total number of persons living in the household; Housing Satisfaction Self-reported satisfaction with current housing situation on a 0–10 scale; Health Restrictions Household Number of household members with health-related limitations (sum of indicators); Degree of urbanization based on official register data (city, suburb, rural area).						
	Control) Age (years), sex (male/female/diverse), education (Highest school degree based on standardized German school system categories), household income (net), and marital status (single/married/divorced/widowed).						
